# MPCTrans: Multi-Perspective Cue-Aware Joint Relationship Representation for 3D Hand Pose Estimation via Swin Transformer

**DOI:** 10.3390/s24217029

**Published:** 2024-10-31

**Authors:** Xiangan Wan, Jianping Ju, Jianying Tang, Mingyu Lin, Ning Rao, Deng Chen, Tingting Liu, Jing Li, Fan Bian, Nicholas Xiong

**Affiliations:** 1 School of Computer Science and Technology, Hubei Business College, Wuhan 430079, China; xianganwan@hbc.edu.cn (X.W.); tjyharry@hbc.edu.cn (J.T.); raoning@hbc.edu.cn (N.R.); tingtingliu89619@gmail.com (T.L.); lijing@hbc.edu.cn (J.L.); bianfanhs@hbc.edu.cn (F.B.); nicholas.xiong@ccnu.edu.cn (N.X.); 2 Hubei Province Key Laboratory of Intelligent Robot, Wuhan Institute of Technology, Wuhan 430079, China; dchen@wit.edu.cn

**Keywords:** depth image, 3D hand pose estimation, multi-perspective cues, Swin Transformer, deep learning

## Abstract

The objective of 3D hand pose estimation (HPE) based on depth images is to accurately locate and predict keypoints of the hand. However, this task remains challenging because of the variations in hand appearance from different viewpoints and severe occlusions. To effectively address these challenges, this study introduces a novel approach, called the multi-perspective cue-aware joint relationship representation for 3D HPE via the Swin Transformer (MPCTrans, for short). This approach is designed to learn multi-perspective cues and essential information from hand depth images. To achieve this goal, three novel modules are proposed to utilize features from multiple virtual views of the hand, namely, the adaptive virtual multi-viewpoint (AVM), hierarchy feature estimation (HFE), and virtual viewpoint evaluation (VVE) modules. The AVM module adaptively adjusts the angles of the virtual viewpoint and learns the ideal virtual viewpoint to generate informative multiple virtual views. The HFE module estimates hand keypoints through hierarchical feature extraction. The VVE module evaluates virtual viewpoints by using chained high-level functions from the HFE module. Transformer is used as a backbone to extract the long-range semantic joint relationships in hand depth images. Extensive experiments demonstrate that the MPCTrans model achieves state-of-the-art performance on four challenging benchmark datasets.

## 1. Introduction

Hand pose estimation (HPE) has applications in various fields, including virtual reality/augmented reality, sign language recognition, and human–computer interaction (HCI) [1]. The goal of HPE is to predict the precise location of each skeletal joint in the hand from a given input image. In the field of HPE, no uniform standard has been established for the number of keypoints in hand models; different datasets define varying numbers of keypoints, with typical models having 14, 16, or 21 keypoints [2,3,4]. Existing methods frequently employ data alignment techniques that transform the input depth image into a canonical space. These methods can be broadly categorized into two primary approaches: (1) traditional methods and (2) learning-based methods. Traditional methods frequently encode hand depth images to extract global features, which are subsequently used to regress 3D joint positions. This process is typically conducted in 2D space, and thus the 3D characteristics of depth images are not adequately captured [5,6,7,8,9]. Alternatively, depth images may be converted into voxel representations, but this procedure demands considerable storage and computational resources [10,11,12,13,14]; depth images may also be aligned in manually defined canonical spaces where performance may be suboptimal [15,16]. By contrast, learning-based methods tend to be more advantageous [17], because they typically achieve data alignment by automatically selecting the most suitable virtual viewpoint.

**Figure 1:**
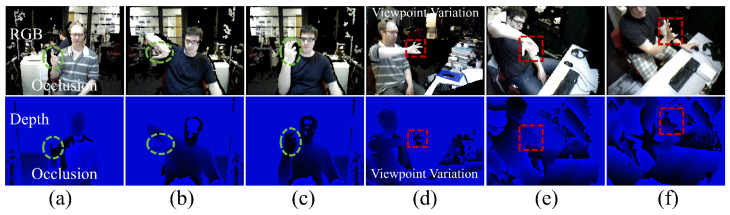
Existing challenges in HPE: (**a**–**c**) serious occlusions and (**d**–**f**) variations in hand appearance from different viewpoints. Some of or even most parts of the hand are missing in these scenarios, resulting in difficulties in HPE. The top line shows RGB images, while the bottom line presents the corresponding depth images.

Despite the superior performance of learning-based methods, they still have several limitations. The approaches described in [18,19] use reinforcement learning to select the optimal viewpoint in a multi-camera system. Cheng et al. [20] proposed to project a single depth image as a point cloud into 3D space, render it into depth maps from specific virtual viewpoints, and then select and fuse informative multiple virtual views. However, in challenging scenarios (Figure 1), such as severe occlusions and extreme viewpoint variations, many crucial parts of the hand may be obscured, resulting in existing methods still falling short of delivering satisfactory results. Thus, leveraging the visible regions of the hand and their geometric interconnections is crucial for achieving accuracy and robustness in predictions. Furthermore, when specific hand parts are obscured from the current viewpoint, relationships from alternate perspectives can be employed to compensate.

**Figure 2:**
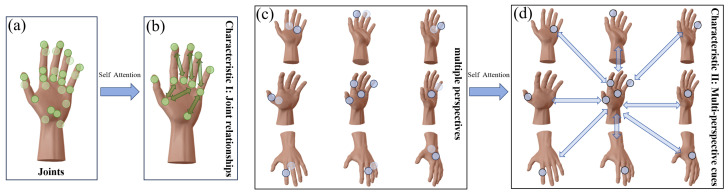
Two key characteristics are revealed from the observation of hand images. Characteristic I: (**a**,**b**) represent the inherent relationships between hand joints. Characteristic II: (**c**,**d**) indicate the multi-perspective cues of the hand. These relationships and cues can be computed using a self-attention mechanism.

To improve HPE by leveraging hand and perspective information, we meticulously observed two key characteristics of the hand. Characteristic I: Hands are frequently used parts of the human body and frequently occluded by themselves or other objects. Nevertheless, inherent relationships between hand joints can provide valuable information for determining hand pose (Figure 2b), even when parts of the hand are hidden or missing. For example, as shown in Figure 1b, if the thumb is obscured, the geometric relationships among the remaining joints, such as those of the index, middle, and pinky fingers, can still help infer the overall hand pose. Characteristic II: Hands can assume various poses and are frequently seen from multiple perspectives due to their high flexibility. We observe that when the current perspective is suboptimal for HPE (e.g., Figure 1f), potential relationships from other perspectives can provide crucial supplementary information (Figure 2d). This observation demonstrates that incorporating multi-perspective cues enhances accuracy.

These findings can be summarized as identifying relationships among hand joints and leveraging multi-perspective cues in hand images. In particular, this study emphasizes the significance of recognizing multi-perspective cues, enabling precise localization and keypoint prediction even under severe occlusion and extreme viewpoints. Therefore, effectively utilizing the two insights is crucial for improving the accuracy of HPE. Motivated by this condition, our work aims to develop a multi-perspective cue method that can identify the relationships among hand joints and the multi-perspective cues of the hand. We propose an adaptive virtual multi-viewpoint (AVM) module that adaptively adjusts virtual viewpoints to capture optimal multi-perspective cues. In addition, we introduce a virtual viewpoint evaluation (VVE) module to evaluate the relevance of each viewpoint and to explore joint relationships within each view. To further utilize hierarchical features, we develop a hierarchy feature estimation (HFE) module to estimate key hand points. Combining these three modules enables our multi-perspective cue-aware joint relationship representation for 3D HPE via the Swin Transformer (MPCTrans) model to be aware of multi-perspective cues in depth hand images, thereby improving the accuracy of HPE.

Overall, our major contributions are summarized as follows:

- An efficient model, namely, MPCTrans, has been developed to leverage optimal multiple-perspective cues for accurate HPE. To the best of our knowledge, this study is the first to apply global adaptive learning to viewpoints centered around the spherical surface of hand point clouds in 3D HPE.
- Three novel modules are proposed to utilize global articulated cues and multi-scale information from hand depth images. In particular, the AVM module adaptively generates ideal virtual multi-view depth images, the HFE module locates and predicts keypoints in the adapted virtual multi-view depth images, and the VVE module evaluates each adapted virtual viewpoint. Combined, these modules fully utilize the information from 3D hand depth images to achieve more accurate 3D HPE.
- Experiments are conducted on four challenging benchmark datasets. The proposed MPCTrans model demonstrates superior performance compared with several existing state-of-the-art methods, thereby confirming its effectiveness.

The remainder of the article is organized as follows: Section 2 reviews related works on 3D HPE and the use of Transformers in this domain. Section 3 provides a comprehensive description of our proposed model. Section 4 presents the experimental results, discusses the four datasets used, and describes the extended experiments. Finally, Section 5 concludes the study.

## 2. Related Work

### 2.1. Three-Dimensional Hand Pose Estimation

Three-dimensional HPE is a persistent and highly researched issue in the field of computer vision. The prevailing approach involves methods based on convolutional neural networks (CNNs). The two primary types of input data for these methods are (1) depth images and (2) RGB images. Our method relies on depth images, and thus, the following discussion focuses on 3D HPE techniques that utilize depth images as input. Among various depth-based approaches, traditional 2D CNN-based methods [2,21,22,23,24,25] are widely used. However, these methods frequently exhibit a simple architecture, and thus capturing the spatial relationships and 3D structure of the hand is challenging. Moreover, these methods are dependent on camera perspectives. Three-dimensional CNN-based methods [11,26] overcome these limitations by employing 3D voxel representations of depth images to encode volumetric information. Although 3D CNN-based methods improve the accuracy of HPE, they require substantial storage capacity and computational power.

By contrast, PointNet-based methods [10,12,16,27,28] transform depth images into point clouds by multiplying depth data by the camera’s intrinsic matrix. Point clouds accurately represent 3D structures, and they are used to estimate joint coordinates in 3D space. PointNet [29] is a deep learning architecture that is capable of processing irregular point cloud data. The first application of PointNet for HPE was HandPointNet [16]. HandFoldingNet [12] later introduced a folding mechanism that converted a predefined hand skeleton into poses, thereby enhancing estimation accuracy. However, the processing of point clouds requires significant computational effort. To mitigate this, existing methods frequently use sparse point clouds to reduce computational load. However, this measure limits their performance.

A new research direction has emerged recently, exemplified by the work in [30], which used diffusion models to predict 3D hand poses. Diffusion models [31,32] recover the original observed data distribution from perturbed data by iteratively denoising at each perturbation step, gradually removing noise.

The closest work to ours is the Virtual View Selection (VVS) model proposed in [20], which also recognizes that input views may not always be ideal. The VVS model employs a learnable module to identify optimal virtual viewpoints for representing point clouds and integrates the estimated poses from these viewpoints. However, virtual viewpoints are chosen from a predefined set of fixed positions, restricting the selection of truly optimal viewpoints. In addition, that method cannot effectively capture the relationships between hand joints due to its reliance on a CNN-based architecture. By contrast, our MPCTrans model imposes no restrictions on virtual viewpoint positions and does not rely on confidence-based selection. Instead, it adaptively learns the optimal virtual viewpoints to capture multi-perspective cues in hand images. By leveraging the Transformer’s nonlocal architecture, our method more effectively captures long-range semantic joint relationships of the hand.

### 2.2. Transformer in 3D Hand Pose Estimation

Here, we review the application of Transformer in 3D HPE because of its relevance to our work. The Transformer model [33], which is an attention-based encoder–decoder framework, has revolutionized the natural language processing (NLP) field. Motivated by these advancements, the computer vision (CV) field has increasingly adopted the Transformer model to advance various CV tasks, including classification, segmentation, and detection, and to process various sensory data streams, such as images, vision-language, and point cloud data [34,35].

The Transformer’s strong capability to capture nonlocal features makes it particularly advantageous for 3D HPE. Consequently, many studies [36,37,38,39,40,41] have introduced the Transformer into this field. Lin et al. [36] employed a Transformer to reconstruct 3D human pose and mesh vertices from a single image. Hand-Transformer [37] introduced a non-autoregressive structured decoding mechanism within the Transformer architecture. Lin et al. [38] presented a graph-convolution-reinforced Transformer that combined graph convolutions with self-attention to model both local and global interactions. A2J-Transformer [41] enhanced A2J [7] by incorporating strong local–global awareness, effectively capturing fine details of interacting hands and the global articulated relationships among joints. Although these existing Transformer-based methods have achieved impressive results, they perform poorly under severe viewpoint variation or occlusion.

## 3. Proposed MPCTrans Model

### 3.1. Overview

**Figure 3:**
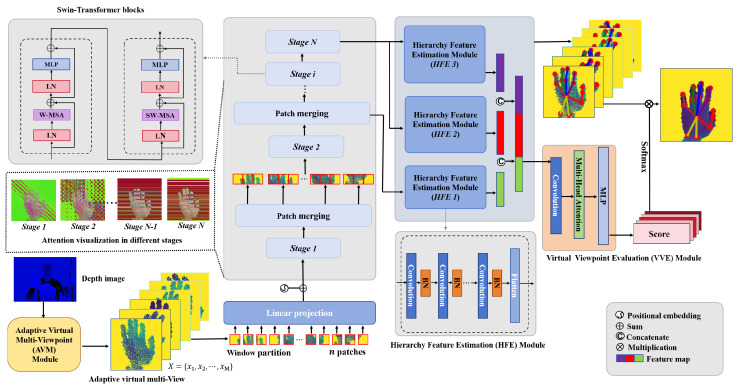
Overview of the MPCTrans model. First, hand depth images are converted into 3D point clouds via the AVM module, which employs adaptive learning at virtual viewpoints to generate optimal virtual multi-view depth images. Second, these adaptive virtual multi-view depth images are partitioned into windows, with attention mechanisms applied only within these partitions. Linear projection and position embedding techniques are utilized to transform patches into 1D vectors. Third, three HFE modules leverage information from lower layers and the output features from the final stage to estimate hand poses for each view. Fourth, the feature maps from the three HFE modules are concatenated. Subsequently, the VVE module assesses this concatenated feature map to assign a score to each view. Finally, the pose estimates and results from each view are fused to produce the final hand pose prediction.

The pipeline of the MPCTrans model is illustrated in Figure 3. The proposed MPCTrans model consists of four components: the AVM module, the backbone, the HFE module, and the VVE module. The first component, i.e., the AVM module, adaptively generates *M* optimal virtual multi-view depth images. The second component is the backbone of the feature extraction. It captures joint relationships of the hand. In this work, the Swin Transformer [42] was employed as the backbone due to its effectiveness in capturing long-range semantic dependencies in hand depth images, such as dependencies between fingertips and finger roots, by using a shifted windowing scheme. The backbone is further enhanced with dropout layers to mitigate overfitting. The third component, namely, the HFE module, leverages multi-layer and multi-scale information to estimate hand poses accurately. The final component, i.e., the VVE module, assesses the virtual viewpoints and reveals the multi-perspective cues in hand images. Given the pose estimates and evaluation results from *M* views, we can fuse them to obtain the final hand position prediction.

### 3.2. AVM Module

**Figure 4:**
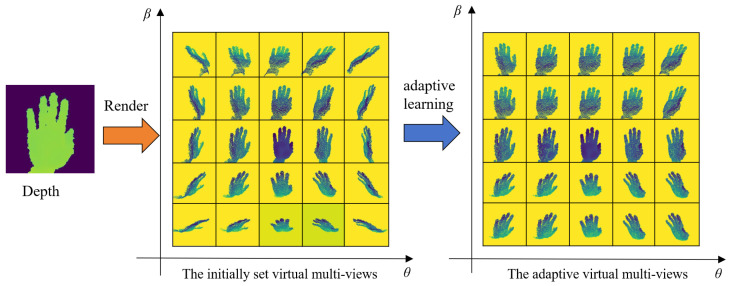
Illustration of the AVM module. Original depth images may not always provide the best perspective for pose estimation. Our method adaptively learns *M* optimal virtual views from *M* initial virtual views, where *M* is set as 25.

We first explain the idea of the AVM module. In [20], the authors demonstrated that dense virtual view sampling improved HPE. Inspired by that study, we hypothesized that our network could further improve the accuracy of HPE if it could learn the ideal virtual gaze point positions from a dense virtual gaze sample. To achieve this goal, we propose the AVM module for 3D HPE. We provide an example to illustrate the optimal viewpoint positions learned by our AVM module for virtual view sampling, as shown in Figure 4.

Large rotations of the viewpoint can cause severe occlusions in depth generation, while small rotations provide only limited performance improvements. Therefore, we uniformly sample *M* points from the azimuth angle within [−60°, 60°] and the zenith angle within [−60°, 60°] on the spherical surface, centered on the hand point clouds, as initial virtual viewpoint positions.

Technically, to enable our network to learn the optimal viewpoint positions for virtual view sampling, we define a learnable angle parameter matrix *Q*, where *Q*exhibits the form $\left[\sqrt{M},\sqrt{M},2\right]$, and the initial values are set to 1. During training, *Q* adaptively learns and records the optimal viewpoint positions for virtual view sampling. We do not directly supervise the AVM Module; instead, we jointly train the AVM Module with our MPCTrans model, which is supervised only on the final hand pose accuracy. Consequently, as the model trains, the learnable matrix parameters *Q* are adaptively adjusted, while the initially defined *M* viewpoints continuously adapt based on the learned matrix parameters *Q* until they converge to optimal positions. The zenith angle and azimuth angles for the adaptive virtual multi-views can be calculated using the following formula:

(1) $$\left[\hat{\theta_{i}},\hat{\beta_{i}}\right]={\hat{Q}}_{i}\left[\theta_{i},\beta_{i}\right],$$

where $\left[\hat{\theta_{i}},\hat{\beta_{i}}\right]$ represents the zenith and azimuth angles of the *i*th adaptive virtual multi-view, ${\hat{Q}}_{i}$ denotes the *i*th learned angle parameter matrix, and $\left[\theta_{i},\beta_{i}\right]$ indicates the zenith and azimuth angles of the *i*th initialized virtual multi-view.

### 3.3. Feature Map Generation

The Swin Transformer [42] uses a hierarchical approach for feature extraction, allowing different layers to capture various receptive fields while computing self-attention. This enables a better use of global information and long-range semantic relationships in hand depth images. Therefore, we use the Swin Transformer as the backbone to generate feature maps from the input virtual multi-views.

In particular, the input adaptive multi-view representations, denoted as $x\in R^{H\times W\times C}$, are first divided into *n* non-overlapping patches, which are subsequently projected onto 1D vectors. Transformer layers are permutation-invariant to the input patch sequences; thus, positional embeddings are necessary to encode the spatial positions and relationships among the patches. These patches, augmented with positional embeddings, are then processed through Swin Transformer blocks. The Swin Transformer block is constructed by replacing the standard multi-head self-attention (MSA) module in a traditional Transformer block with a shifted-window-based attention mechanism. In our implementation, the window size was set to 11. Each Swin Transformer block is calculated as follows:

(2) $$\left\{\begin{array}{c} w^{f}=MLP\left[LN(w^{f})\right]+w^{f}\\ {\hat{w}}^{f}=W\text{-}MSA\left[LN(w^{f-1})\right]+w^{f-1}\\ w^{f+1}=MLP\left[LN(w^{f+1})\right]+w^{f+1}\\ {\hat{w}}^{f+1}=SW\text{-}MSA\left[LN(w^{f})\right]+w^{f} \end{array},\right.$$

where W-MSA and SW-MSA represent the window-based MSA and the shifted-window partitioning configurations in the Swin Transformer, respectively. ${\hat{w}}^{f}$ and $w^{f}$ represent the output patch vectors from the (S)W-MSA and MLP modules of the Transformer block *f*, respectively. LN denote layer normalization, and MLP refers to multiple fully connected layers.

The Transformer blocks are divided into *N* stages through patch merging layers. The lower-layer and output features from the final stage are then fed into the HFE modules to estimate the pose for each view. In contrast with the raw Swin Transformer, we only perform 4× and 8× downsampling and forego 16× downsampling to preserve fine spatial information and reduce model size.

### 3.4. HFE Module

To effectively use important information from feature maps for hand pose c, we place anchor points uniformly within the feature maps. This study uses three HFE modules to examine the relationship between each anchor point and the hand joints. Each HFE module uses CNNs [43,44] to extract features, with the output of each convolution layer normalized using a BatchNorm layer. Finally, these features are flattened to estimate hand pose.

The HFE1 module takes the features from the second patch merging layer as input and predicts the anchor weight $G_{j}^{i}\left(a\right)$ for each anchor point toward the joint in each view. The HFE2 and HFE3 modules use features from the final stage to predict the 2D plane offset $O_{j}^{i}\left(a\right)$ and the depth value $D_{j}^{i}\left(a\right)$ of each anchor point toward the joint in each view, respectively. The anchor weight of each anchor point toward joint *j*, the 2D plane position of joint *j*, and the depth of joint *j* in each view can be calculated as follows:

(3) $$\left\{\begin{array}{c} {\tilde{G}}_{j}^{i}\left(a\right)=\frac{e^{G_{j}^{i}\left(a\right)}}{\sum_{a\in A}e^{G_{j}^{i}\left(a\right)}}\\ {\hat{H}}_{j}^{i}=\sum_{a\in A}{\tilde{G}}_{j}^{i}\left(a\right)\left(H^{i}\left(a\right)+O_{j}^{i}\left(a\right)\right)\\ {\hat{D}}_{j}^{i}=\sum_{a\in A}{\tilde{G}}_{j}^{i}\left(a\right)D_{j}^{i}\left(a\right) \end{array},\right.$$

where *a* and *A* represent an anchor point and the set of anchor points, respectively. ${\tilde{G}}_{j}^{i}\left(a\right)$ is obtained via softmax calculation, and it can be considered as the normalized anchor weight of anchor point *a* toward joint *j* in the *i*th adaptive virtual multi-view across all anchor points. ${\hat{H}}_{j}^{i}$ and ${\hat{D}}_{j}^{i}$ represent the estimated 2D plane position and depth value of joint *j* in the *i*th adaptive virtual multi-view, respectively. $H^{i}\left(a\right)$ indicates the 2D plane position of the anchor point *a* in the *i*th adaptive virtual multi-view.

Each perspective contributes differently to HPE, and thus we concatenate the features obtained from the three HFE modules and then feed them into the VVE module to evaluate each virtual viewpoint.

### 3.5. VVE Module

To evaluate the importance of each perspective, we use the VVE module to evaluate the score for each view. Instead of directly monitoring these results, we base our monitoring on the final hand pose accuracy. We do not select these views based on their ratings; instead, we use all views as input for the backbone because each view represents an ideal perspective obtained through adaptive learning.

**Figure 5:**
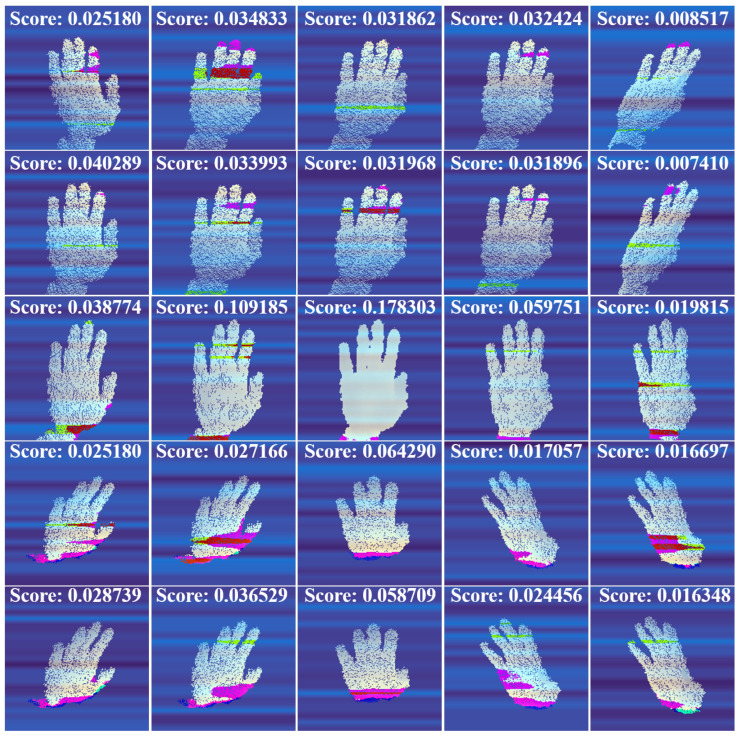
Attention visualization of different perspectives in the VVE module. Each perspective contributes differently to HPE, resulting in different attention visualizations in the multi-head attention of the VVE module. Consequently, each perspective has a different rating.

To reduce computational cost, the output of the three HFE modules is concatenated and fed into the high-level function. The VVE module employs CNNs to extract features and applies multi-head attention [6] to merge the multi-view features. An MLP layer then maps the features of each view to a scalar value. As shown in Figure 5, each perspective contributes differently to the final hand pose prediction, resulting in different results.

The scalar score derived from each view, computed via the multi-head attention mechanism, is employed to perform a weighted summation of the corresponding joint positions across multiple virtual viewpoints, yielding the estimated position of joint *j*, denoted as ${\hat{J}}_{j}$. Finally, we merge the results with the estimated hand poses to obtain the final prediction $\hat{J}$. The estimated joint *j* and the overall prediction can be calculated using the following formulas:

(4) $$\left\{\begin{array}{c} {\hat{J}}_{j}=\sum_{i=1}^{M}s^{i}\left(R^{i}\left[{\hat{H}}_{j}^{i},{\hat{D}}_{j}^{i}\right]+t^{i}\right)\\ \hat{J}=\sum_{i=1}^{M}s^{i}\left(R^{i}\left[{\hat{H}}^{i},{\hat{D}}^{i}\right]+t^{i}\right) \end{array},\right.$$

where ${\hat{J}}_{j}$ is the estimated joint *j*. $\hat{J}$ represents the final predicted result for all hand joints. $s^{i}$ is the score of the *i*th adaptive virtual multi-view after applying softmax, and $\left[R^{i},t^{i}\right]$ denotes the extrinsic camera parameters for the *i*th adaptive virtual multi-view. ${\hat{H}}^{i}$ and ${\hat{D}}^{i}$ indicate the estimated 2D plane position and depth value of the *i*th adaptive virtual multi-view, respectively. They can be calculated using the following formula:

(5) $$\left\{\begin{array}{c} {\hat{H}}^{i}=\left[{\hat{H}}_{1}^{i},{\hat{H}}_{2}^{i},{\hat{H}}_{3}^{i},\ldots ,{\hat{H}}_{k}^{i}\right]\\ {\hat{D}}^{i}=\left[{\hat{D}}_{1}^{i},{\hat{D}}_{2}^{i},{\hat{D}}_{3}^{i},\ldots ,{\hat{D}}_{k}^{i}\right] \end{array},\right.$$

where *k* is the total number of hand joints.

### 3.6. Loss Functions

To train the MPCTrans model, we employed three loss functions: (1) joint estimation, (2) anchor weight, and (3) joint score losses.

Joint estimation loss: We supervised the final output by using the joint estimation loss, which was formulated as follows:

(6) $$loss_{1}=\lambda \sum_{j\in J}L_{\tau_{1}}\left({\hat{H}}_{j}-H_{j}\right)+\sum_{j\in J}L_{\tau_{2}}\left({\hat{D}}_{j}-D_{j}\right),$$

where λ was set to 0.5 to balance the 2D plane offset and depth estimation tasks. J denotes the set of hand joints. ${\hat{H}}_{j}$ and ${\hat{D}}_{j}$ represent the estimated 2D plane position and depth value of joint *j* from Equation (4). $H_{j}$ and $D_{j}$ are the target 2D plane and depth positions of the joint *j*, respectively. $L_{\tau }\left(\cdot \right)$ is the smooth L1 loss function [45], which is defined as

(7) $$L_{\tau }\left(x\right)=\left\{\begin{array}{cc} \frac{1}{2\tau }x^{2}, & \mathrm{for}\;|x|<\tau ,\\ \left|x\right|-\frac{\tau }{2}, & \mathrm{otherwise}. \end{array}\right.$$

In Equation (6), $\tau_{1}$ was set to 1 and $\tau_{2}$ was set to 3.

Anchor weight loss: To select informative anchor points around the joints and improve the generalization ability of our model, we used the anchor weight loss, which was given by

(8) $$loss_{2}=\sum_{j\in J}L_{\tau_{1}}\left(\sum_{a\in A}{\tilde{G}}_{j}\left(a\right)H\left(a\right)-H_{j}\right).$$

Joint score loss: We trained the VVE module with the other modules and monitored the final hand position accuracy. The joint score loss was calculated as follows:

(9) $$loss_{3}=\sum_{n=1}^{K}L_{\tau_{1}}\left({\hat{J}}_{j}-J_{j}\right),$$

where ${\hat{J}}_{j}$ is the estimated joint *j* from Equation (4), $J_{j}$ is the ground-truth position of joint *j*, and *K* is the total number of hand joints.

Finally, the total loss function was defined as follows:

(10) $$totalloss=\gamma_{1}loss_{1}+\gamma_{2}loss_{2}+\gamma_{3}loss_{3},$$

where $\gamma_{1}$,$\gamma_{2}$, and $\gamma_{3}$ were set to 1, 3, and 0.1, respectively, to balance the three losses.

## 4. Experiments

### 4.1. Datasets and Evaluation Metric

ICVL: The ICVL dataset [3] includes 22,000 training depth images and two test sequences with 800 depth images each. The training images were collected from 10 subjects, while the test images were collected from 2 subjects. Each hand pose annotation includes 16 joints.

NYU: The NYU dataset [2] contains RGBD data that comprise 8252 test images and 72,757 training images. The training set was collected from a single subject, while the test set includes data from two subjects. Each frame contains RGBD data captured using three Kinect devices: a front view and two side views. For training, we only used depth data from the front view. A total of 36 hand joints are annotated. We used 14 of the 36 joints for evaluation based on previous studies [2,20].

Hands19-Task1: The Hands19-Task1 dataset [46] is based on the BigHand2.2M benchmark dataset [4]. It comprises 175,000 training images from five subjects and 125,000 test images from ten subjects, with an overlap of five subjects between the test and training sets. This dataset is highly challenging due to its exhaustive coverage of viewpoints and articulations.

MSRA: The MSRA dataset [47] contains 76,000 depth images collected from nine participants, with each participant performing 17 distinct gestures, and approximately 500 frames recorded per gesture. Each annotated hand pose consists of 21 joints.

We used the following metrics suggested in [3] for our quantitative evaluations.

Percentage of successful frames across various error thresholds: This metric indicates the proportion of test images in which the error for every joint remains within a specified threshold distance from the ground truth. A frame is deemed successful only when all joint errors are below the designated threshold.

Mean joint error: This metric represents the average Euclidean distance, measured in millimeters, between the ground truth and predicted joint positions across all test images.

### 4.2. Implementation Details

The proposed MPCTrans model was implemented as follows. First, input depth images from the datasets were cropped and resized to 176 × 176 pixels. For data augmentation, the cropped depth maps were randomly scaled while generating the multi-view depth. The MPCTrans model was trained for 100 epochs with a batch size of eight for computational efficiency. Intermediate weights were initialized from the Swin Transformer base backbone pre-trained on ImageNet-22k [48]. The Adam optimizer was employed with an initial learning rate of 0.0001, which decayed by 0.95 per epoch. All experiments were conducted using three Nvidia RTX 3090 Ti GPUs and the PyTorch framework.

### 4.3. Experimental Results

We compared the MPCTrans model with state-of-the-art approaches by using the ICVL, NYU, MSRA, and Hands19-Task1 datasets. These approaches included methods that used 2D depth images as input, such as DeepPrior++ [5], DenseReg [6], A2J [7], AWR [9], NARHT [37], Rokid [8], Virtual View Selection (VVS) [20], A2j-transformer [41], BT [49], IPR [50], MuTr [51], and TriHornNet [14], and methods that used 3D representations of depth maps as input, including HandPointNet [16], Point-to-Point [10], HandFoldingNet [12], V2V [11], IPNet [13], HandVoxNet [52], HandVoxNet++ [53], and HandDiff [30].

We first compared the MPCTrans model with state-of-the-art techniques by using the ICVL, MSRA, and NYU datasets. The details and results of these techniques were obtained from [20,30]. Table 1 presents the mean 3D joint error. The results indicate that, in terms of mean 3D joint error, the MPCTrans model achieved superior prediction accuracy compared with existing state-of-the-art methods on the NYU, MSRA, and ICVL datasets.

From Table 1, it is evident that on the NYU dataset, the MPCTrans model achieved the lowest mean 3D joint error of 6.32 mm. Compared to the transformer-based models NARHT (9.8 mm) and A2j-transformer (8.43 mm), our model achieved error reductions of 35% and 25%, respectively, by more effectively capturing hand joint movements. It also outperformed HandDiff (7.38 mm) and TriHornNet (7.68 mm) by a significant margin and slightly surpassed VVS (6.40 mm). These results demonstrated the effectiveness of our model in handling occlusions and viewpoint variations when perceiving multi-view cues. Furthermore, on the ICVL dataset, MPCTrans achieved the lowest mean joint error (4.66 mm) among all competing methods. Additionally, on the MSRA dataset, MPCTrans achieved the lowest error of 6.26 mm, further demonstrating the robustness of our model in maintaining high accuracy across diverse datasets.

**Figure 6:**
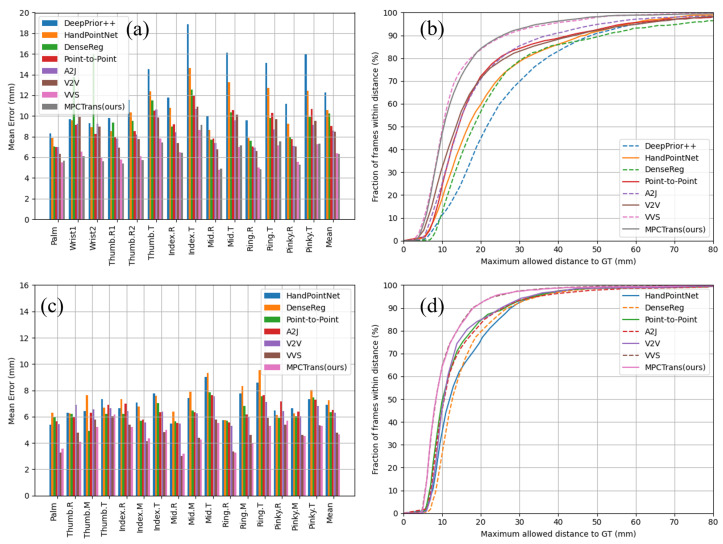
Comparison of the MPCTrans model with state-of-the-art methods (DeepPrior++ [5], HandPointNet [16], DenseReg [6], Point-to-Point [10], A2J [7], V2V [11], VVS [20]) on the NYU and ICVL datasets. (**a**,**b**) Mean joint error per hand joint and percentage of successful frames over different error thresholds in the NYU dataset. (**c**,**d**) Mean joint error per hand joint and percentage of successful frames across various error thresholds in the ICVL dataset.

Figure 6 illustrates the percentage of successful frames across various error thresholds, and the mean joint error for each hand joint on the NYU and ICVL datasets. We compared the MPCTrans model with several representative approaches. The results clearly demonstrate that the MPCTrans model achieved lower mean joint errors for most hand joints compared with state-of-the-art methods. In addition, the MPCTrans model exhibited superior performance in terms of the percentage of successful frames across various error thresholds, outperforming state-of-the-art methods across most ranges of maximum allowable distance from the ground truth. On the NYU dataset, Figure 6a demonstrates that our method achieved lower prediction errors across most joints, particularly for the wrist and thumb joints, compared with other methods. As shown in Figure 6b, with an increase in the maximum allowable distance, our model achieved higher success rates across most error thresholds. Notably, at the 30 mm and 50 mm thresholds, the proportion of successful frames surpassed that of existing methods like A2j and V2V. These results indicate that MPCTrans not only excelled in reducing absolute error but also demonstrated stronger robustness in handling complex hand gestures. On the ICVL dataset, Figure 6c,d reveal similar trends. Although the ICVL dataset primarily consists of standard hand poses, MPCTrans achieved the lowest mean joint error across most joints, while maintaining a high proportion of successful frames at various error thresholds. This demonstrates that our model effectively leveraged multi-scale information to generate highly accurate pose estimations under optimal conditions.

In addition, we compared the MPCTrans model with several representative state-of-the-art approaches on the Hands19-Task1 dataset. Table 2 provides the mean joint error of these methods. Consistent with the results from previous experiments, our model achieved superior prediction accuracy in terms of mean joint error compared with state-of-the-art approaches on the Hands19-Task1 dataset.

Overall, our MPCTrans model exhibited varying performance improvements on the NYU, Hands19-Task1, MSRA, and ICVL datasets compared with state-of-the-art methods. These results demonstrate that our model effectively identified optimal viewpoints and generated virtual multi-view depth images, surpassing the limitations of physical sensors. Furthermore, by leveraging multi-view cues, the model accurately predicted hand keypoints from the virtual depth images and assessed the reliability of each virtual viewpoint, ensuring that only the most informative views contributed to the final pose estimation.

### 4.4. Ablation Study and Analysis

Component effectiveness analysis: To evaluate the effectiveness of each component of the MPCTrans model, we performed experiments on the ICVL dataset, focusing on three parts: (1) the AVM module, (2) Swin Transformer, and (3) the VVE module. The specific implementation details are defined as follows: (1) directly estimating 3D hand joints without the AVM module, (2) replacing Swin Transformer with ResNet-50 [54], (3) directly regressing 3D hand joints without the VVE module. The results are presented in Table 3.

Table 3 indicates the following: (1) Removing the AVM module reduced the distance by 0.6 mm, further proving the effectiveness of the AVM module in our method. (2) Replacing the Swin Transformer with ResNet-50 resulted in a performance drop of 0.11 mm, demonstrating that our HFE module performs better with the Transformer architecture. (3) Removing the VVE module reduced performance by 0.23 mm, indicating that virtual multi-views require the VVE module to balance the score of each perspective.

**Figure 7:**
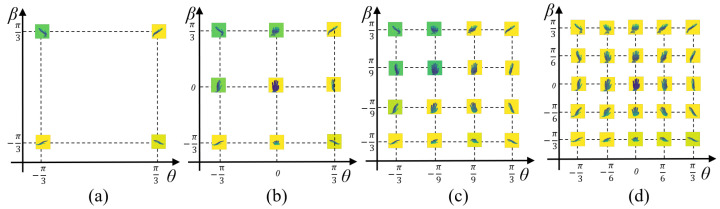
Viewpoint initialization scheme. (**a**), (**b**), (**c**), and (**d**) represent the initial virtual viewpoint positions for 4, 9, 16, and 25 adaptive virtual multi-views, respectively.

Effect of adaptive virtual multi-view number: To study the influence of the number of adaptive virtual multi-views on the MPCTrans model, we initialized 4, 9, 16, and 25 points as initial virtual viewpoints for the adaptively sampled virtual views. We compared their performance on the NYU, ICVL, and Hands19-Task1 datasets. The setup for different initial virtual viewpoint positions is shown in Figure 7.

**Figure 8:**
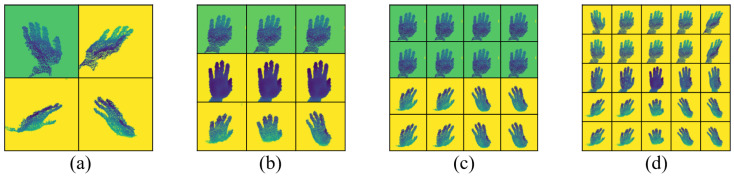
Visualization of different numbers of adapted virtual multi-views: (**a**), (**b**), (**c**), and (**d**) represent the 4, 9, 16, and 25 adapted virtual multi-views, respectively, learned from the initial virtual viewpoint positions shown in Figure 7.

Figure 8 shows the adapted virtual multi-views obtained through adaptive learning from the initial virtual viewpoint positions presented in Figure 7.

Table 4 indicates that the performance of our method degraded when only an adaptive virtual view was used. This result is likely due to the MPCTrans model requiring multiple adaptive virtual views for effective training and convergence. Using only a single view reduced performance within the network. Furthermore, even with only four adaptive virtual multi-views, our method still achieved competitive performance. We observed that the error in HPE decreased as the number of multi-views increased. Thus, increasing the number of views improved HPE performance.

**Figure 9:**
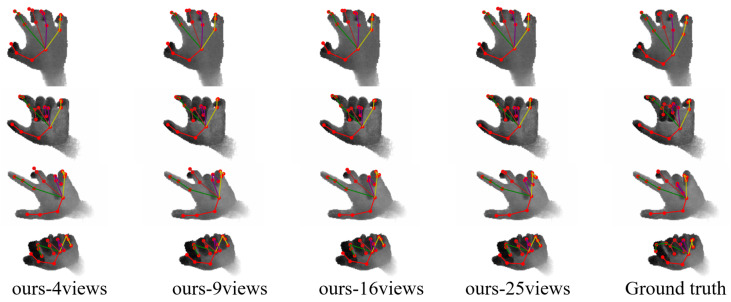
Comparison of the visualization results of the MPCTrans model with different numbers of adaptive virtual multi-views on the ICVL dataset. “ours-4views”, “ours-9views”, “ours-16views”, and “ours-25views” represent the results of the model with 4, 9, 16, and 25 adaptive virtual multi-views, respectively.

Figure 9 visualizes the results of the MPCTrans model with different numbers of adaptive virtual multi-views on the ICVL dataset. To make viewing joints and their connections easier, the hands in these illustrations are shown in grayscale. As the number of views increased, our method became more accurate in predicting the occluded areas of the hand. This improvement is particularly noticeable in the first and third rows.

**Figure 10:**
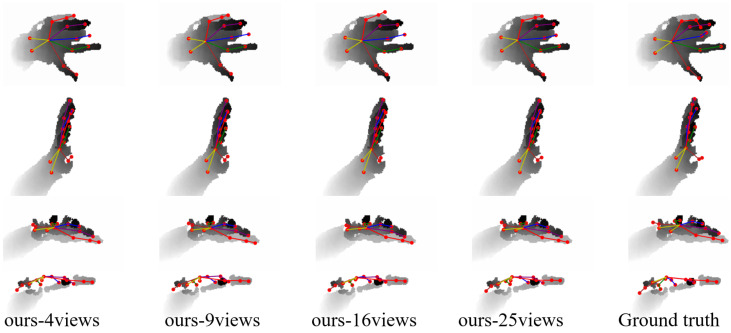
Comparison of the visualization results of the MPCTrans model with different numbers of adaptive virtual multi-views on the NYU dataset.

Figure 10 visualizes the results of the MPCTrans model with different numbers of views on the NYU dataset. The visualization results indicate that using 25 views provided more accurate estimates of wrists and joints in areas with missing depth values compared with other view counts.

Effect of the backbone: To investigate the impact of different backbone networks on the performance of our model, we conducted experiments on the MSRA dataset using three commonly used backbones: ResNet, ViT [55], and Swin Transformer.

Table 5 presents the mean joint error. The results indicate that the Swin Transformer achieved the lowest error (6.26 mm), outperforming CResNet (6.87 mm) and ViT (6.51 mm). Both the Swin Transformer and ViT outperformed ResNet, primarily due to the superior ability of Transformers to encode semantic visual information compared to CNNs. Furthermore, the Swin Transformer demonstrates the best performance, confirming that it can effectively process multi-view cues and multi-scale information from complex hand poses when used as the backbone of our model.

## 5. Conclusions

In this study, we proposed a multi-perspective cue-aware joint relationship representation for HPE, called MPCTrans, which adaptively learned multi-perspective cues. Our method captured long-range semantic joint relationships of the hand by leveraging the Transformer’s nonlocal architecture. In addition, each virtual view was evaluated, and these evaluations were combined with the corresponding estimated poses to achieve more accurate 3D hand position estimations. Experiments on four challenging benchmark datasets demonstrated the effectiveness of the proposed method. In our future work, we aim to further enhance our approach, particularly by extending it to real-time estimation.

## Figures and Tables

**Table 1:** Comparison of mean 3D joint error, measured in millimeters, with state-of-the-art methods on the ICVL, MSRA, and NYU datasets.

Methods	Source	NYU	ICVL	MSRA
DeepPrior++ [5]	ICCVW(2017)	12.24	8.1	9.5
HandPointNet [16]	CVPR (2018)	10.54	6.93	8.5
V2V [11]	CVPR (2018)	8.42	6.28	7.59
DenseReg [6]	CVPR (2018)	10.21	7.24	7.2
Point-to-Point [10]	ECCV (2018)	9.10	6.3	7.7
A2J [7]	ICCV (2019)	8.61	6.46	-
AWR [9]	AAAI (2020)	7.37	5.98	7.2
NARHT [37]	ECCV (2020)	9.8	6.47	7.55
HandFolding [12]	ICCV (2021)	8.58	5.95	7.34
VVS [20]	AAAI (2022)	6.40	4.79	-
IPNet [13]	AAAI (2023)	7.17	5.76	6.92
A2j-transformer [41]	CVPR (2023)	8.43	-	-
TriHornNet [14]	ESWA (2023)	7.68	5.73	7.13
HandDiff [30]	CVPR (2024)	7.38	5.72	6.53
MPCTrans (Ours)	-	**6.32**	**4.66**	**6.26**

Note: The bold values indicate the best performance among the compared methods.

**Table 2:** Comparison with state-of-art approaches on the Hands19-Task1 dataset.

Methods	Mean Error (mm)
BT [49]	23.62
IPR [50]	19.63
MuTr [51]	15.64
V2V [11]	15.57
HandVoxNet [52]	15.57
AWR [9]	13.76
A2J [7]	13.74
Rokid [8]	13.66
HandVoxNet++ [53]	13.35
VVS [20]	12.55
MPCTrans (Ours)	**12.52**

Note: The bold values indicate the best performance among the compared methods.

**Table 3:** Component effectiveness analysis. × indicates that the module or network was not used, ✓ indicates that it was used.

AVM	Swin Transformer	VVE	Mean Error (mm)
×	✓	✓	5.26
✓	×	✓	4.77
✓	✓	×	4.89
✓	✓	✓	**4.66**

Note: The bold values indicate the best performance.

**Table 4:** Comparison of mean joint error on the NYU, ICVL, and Hands2019-Task1 datasets with different numbers of virtual views.

Number of Views	NYU	ICVL	Hands19-Task1
1	8.69	5.66	14.94
4	6.85	4.85	13.16
9	6.49	4.84	12.71
16	6.48	4.77	12.56
25	**6.32**	**4.66**	**12.52**

Note: The bold values indicate the best performance.

**Table 5:** Effect of backbone.

Backbone	Mean Error (mm)
ResNet	6.87
ViT	6.51
Swin Transformer	**6.26**

Note: The bold values indicate the best performance.

## Data Availability

ICVL https://labicvl.github.io/hand.html (accessed on 29 October 2024); HANDS2019 https://sites.google.com/view/hands2019/challenge (accessed on 29 October 2024); NYU https://jonathantompson.github.io/NYU_Hand_Pose_Dataset.htm (accessed on 29 October 2024).
